# Task-Selective Memory Effects for Successfully Implemented Encoding Strategies

**DOI:** 10.1371/journal.pone.0038160

**Published:** 2012-05-31

**Authors:** Eric D. Leshikar, Audrey Duarte, Christopher Hertzog

**Affiliations:** 1 Department of Psychology, Brandeis University, Waltham, Massachusetts, United States of America; 2 School of Psychology, Georgia Institute of Technology, Atlanta, Georgia, United States of America; The Scripps Research Institute, United States of America

## Abstract

Previous behavioral evidence suggests that instructed strategy use benefits associative memory formation in paired associate tasks. Two such effective encoding strategies–visual imagery and sentence generation–facilitate memory through the production of different types of mediators (e.g., mental images and sentences). Neuroimaging evidence suggests that regions of the brain support memory reflecting the mental operations engaged at the time of study. That work, however, has not taken into account self-reported encoding task success (i.e., whether participants successfully generated a mediator). It is unknown, therefore, whether task-selective memory effects specific to each strategy might be found when encoding strategies are successfully implemented. In this experiment, participants studied pairs of abstract nouns under either visual imagery or sentence generation encoding instructions. At the time of study, participants reported their success at generating a mediator. Outside of the scanner, participants further reported the quality of the generated mediator (e.g., images, sentences) for each word pair. We observed task-selective memory effects for visual imagery in the left middle occipital gyrus, the left precuneus, and the lingual gyrus. No such task-selective effects were observed for sentence generation. Intriguingly, activity at the time of study in the left precuneus was modulated by the self-reported quality (vividness) of the generated mental images with greater activity for trials given higher ratings of quality. These data suggest that regions of the brain support memory in accord with the encoding operations engaged at the time of study.

## Introduction

It is well known that the manner in which information is initially studied has an impact on how that information is subsequently retrieved [1]. Use of encoding strategies at the time of study can facilitate memory for single items and also for associations between items [2]. Two particularly effective associative encoding strategies are sentence generation, where participants actively incorporate two or more words together into a meaningful sentence, and interactive visual imagery, where participants integrate visual tokens of the items into an imagistic representation [2]–[6]. Neuroimaging investigations suggest that regions of the brain activate *selectively* in support of memory (e.g., hits > misses) in accord with the types of cognitive processes engaged at the time of study. For instance, tasks that emphasize perceptual encoding tend to engage perceptual regions while tasks that promote semantic cognitive processes engage language regions, and so forth [7]–[11]. It stands to reason that encoding processes that promote different operations at study, such as visual imagery and sentence generation, would recruit brain regions in support of memory in a *task-selective* manner reflecting the processing demands of each respective task. Although behavioral investigations have established the memory benefits of strategy engagement, relatively less is known about the neural substrates involved in the use of elaboratively rich encoding strategies, and further, how different strategies might be supported by different cortical regions.

Mental imagery and sentence generation emphasize the generation of qualitatively different types of *mediators* (e.g., production of mental images versus sentences) [12]. Both mental imagery production and sentence generation facilitate memory relative to less effective associative strategies such as rote rehearsal [2], [13]. Although multiple fMRI investigations have used either sentence generation or interactive imagery to facilitate memory, few have incorporated both within a single experiment to directly compare functional recruitment associated with each strategy. Without direct comparisons across tasks, it is difficult to truly isolate task-selective memory effects associated with different encoding operations. Those studies that *have* used visual imagery to promote memory for inter-item associations, however, have reported memory-related activity in the precuneus, the middle occipital gyrus and the lingual gyrus [14]–[16]–regions implicated in the generation of visual mental images [17]–[19]. Similarly, studies that have used sentence generation have reported memory related activity in left inferior frontal and lateral temporal regions [16], [20]. Thus, it is expected that task-selective associative memory effects should emerge in regions associated with the processing demands of each respective task.

One of the few studies that has incorporated both visual imagery and sentence generation within the same experimental design observed recollection effects unique to each respective encoding task [16]. In that study, task specific recollection effects for trials eliciting a “remember” judgment, were found at the time of study in the precuneus and lingual gyrus for visual imagery, and in inferior frontal and temporal regions for sentence generation. That investigation, however, did not assess whether participants successfully implemented each respective task for each trial. Behavioral investigations have found that instructed strategies, as measured by self-reports, are not always successfully implemented [21], [22]. That is, despite attempting to produce mediators in paired associate tasks (e.g., a sentence or an image), participants are not always able to do so for every trial. Therefore, an important consideration in studies of encoding strategies is the degree of successful implementation. Multiple fMRI investigations have adopted self-report measures to evaluate the success of performing a specific encoding strategy, but these studies conflated the quality of the generated mediator with task success [14], [15]. In imagery studies, for example, participants rated the quality of the generated mediator (e.g., how vivid was the image you generated?), not whether the participants were successful in carrying out the encoding operation (e.g., did you generate a visual image for this trial?) [14], [15]. Consequently, these studies do not exclusively isolate activity associated with the successful use of a strategy. In the current experiment, we collected self-reports of strategy success (did you successfully generate a sentence/image on this trial?) at the time of study as well as a rating of the quality of the generated mediator in a post-test assessment in order to examine task-selective memory associated with the successful implementation of each respective strategy.

In this experiment, participants studied pairs of abstract words (e.g., justice -truth) under either interactive imagery or sentence generation conditions. We were primarily interested in characterizing task-selective memory effects at the time of study and test associated with each encoding strategy for trials where a mediator was reported as successfully generated. We made three predictions:

First, we predicted task-selective activity in support of associative memory accuracy at the time of *study* for visual imagery and sentence generation, respectively, in accord with the processing demands of each task. Specifically, we predicted activity in middle occipital, precuneus, and lingual gyrus for visual imagery, and left inferior frontal and lateral temporal activations for sentence generation. Such a finding would support the notion that regions of the brain support memory selectively dependent on the operations engaged during study [10], [11]. Further, finding task-selective memory effects associated with each respective strategy would serve to substantiate the self-report measures from behavioral investigations where participants report engaging in qualitatively different types of cognitive processes while producing visual images versus sentences [23].

Second, we predicted task-selective memory retrieval effects in similar brain regions as those noted above at study. Previous work has shown that retrieval effects are dependent on the manner in which the events were initially encoded [9]. For instance, Dobbins and Wagner [8] showed that perceptual regions at the time of *test* supported successful source judgments for items studied with attention to their perceptual detail (is the item bigger/smaller than the previous item?) relative to those items studied for their conceptual properties (is the item living/non-living?). Thus, finding task-selective effects for each respective task would support the notion that regions subserving encoding operations are re-activated at the time of test to support successful remembering [9].

Alternatively, detecting task-selective memory effects at study and test for imagery and sentence generation could be reduced by processing overlap shared by the two encoding strategies. Evidence suggests that semantic knowledge of concrete items automatically evokes perceptual detail [24]. That is, bringing to mind semantic information of concrete objects, such as an apple, often automatically generates images of those objects. To reduce the likelihood of this type of spontaneous imagery engagement, we used abstract words as stimuli for this study. It is possible, however, that selecting and generating mental images of abstract words draws on the same semantic-conceptual processes needed for generating sentences. That is, creating images for abstract items requires semantic processing of the abstract concepts (i.e., “justice”). Engagement in similar semantic processes in both strategies would reduce the likelihood of finding task-selective memory effects for sentence generation.

Third, studies have reported that some brain regions support memory in a domain-general fashion regardless of the mental operations deployed [8], [25], [26]. Hippocampal [20], [27], [28] and prefrontal cortex activations [20], [29] have been detected in previous associative memory investigations. Several theories [30], [31] argue that frontal-hippocampal interactions play a crucial role in the binding of information into an associative memory trace that can be accessed by a controlled retrieval search. Thus, we predicted hippocampal and inferior frontal activations at both study and test in support of associative memory accuracy for both tasks.

## Methods

### Participants

A total of twenty young adults recruited from the Georgia Institute of Technology completed the experimental procedures. Three participants were excluded due to chance performance on the recognition test for either encoding strategy, and an additional participant was excluded due to an insufficient miss (i.e. forgetting) rate, leaving a total of 16 participants (mean age: 24.75, *SD* 4.3, 8 females, range: 18–30) included in this report. Participants were all right-handed native-English speakers with normal or corrected-to-normal vision. No participant reported cardiovascular disease, psychoactive drug use, psychiatric conditions, or neurological disorders (e.g. stroke, epilepsy, multiple sclerosis, etc.). None of the participants were taking CNS-active or vaso-active medications**.** Informed written consent was obtained from all participants prior to participation in accordance with the Institutional Review Board at the Georgia Institute of Technology. The IRB at the Georgia Institute of Technology approved all procedures associated with this study. All participants were paid $10 per hour for their participation.

### Stimuli

A total of 480 abstract nouns taken from the MRC Psycholinguistic database (Coltheart, 1981) served as stimuli. Words were 3–9 letters in length and were constrained to items with a concreteness rating of 217–411 (*M = *326, *SD* = 45). Abstract words were explicitly chosen in this task to minimize spontaneous imagery of the word concept. Stimuli were screened to exclude words with homophones (e.g., “poll” for “pole”) and words with multiple meanings (e.g., minute). At study, 384 words served as stimuli for the word pair trials (192 pairs) while 96 words were presented in single word trials. Words presented as part of a pair or singly were counterbalanced across participants. Words subtended a maximum vertical and horizontal visual angle of approximately 1.1 degrees and 3.8 degrees.

### Procedure

All experimental procedures took place during a single session. Participants were instructed and trained on the study and test phases of the experiment. Training included 16 practice study trials and 8 practice test trials. To ensure task comprehension, participants verbally reported the task instructions back to the experimenter. In preparation for the MRI scans, participants were given noise-dampening earplugs, a 4-button optical response box in their right hand, and MRI-compatible headphones to communicate with the experimenter between runs. Participants were instructed to minimize all movements, especially head movements for the duration of the experiment.

**Figure 1:**
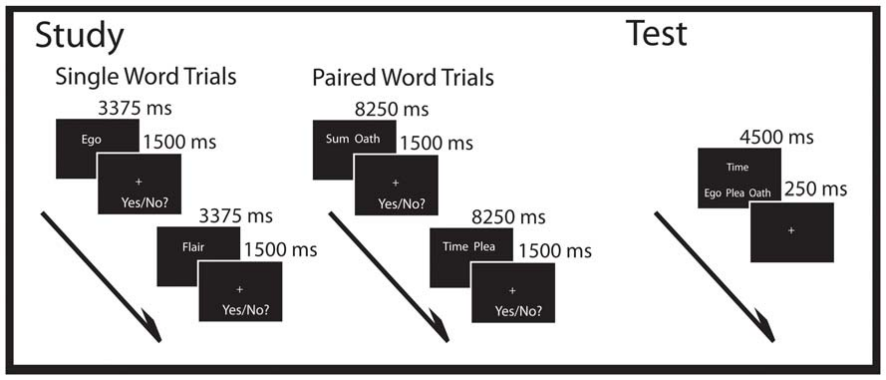
Trial schematic for the study and test phases respectively.

The study phase of the experiment was conducted over 4 scanning runs. A total of 192 word pair trials and 96 single word trials were presented at study. Half of the single and word pairs appeared in each of the two encoding conditions. For each study phase run, 24 word pair and 12 single word trials were displayed per condition. Word pair trials were presented for 9750 ms while the single word trials were presented for 4875 ms. Within a trial, single word and word pair stimuli were presented for 3375 and 8250 ms, respectively, and then participants were given 1500 ms to rate their success in generating a mediator by making a yes/no report regarding the success in implementing the instructed strategy(e.g., visual image or sentence) for that trial (see **Figure 1**). All words were presented in white 36-point Arial font on a black background. To reduce task-switching costs, trials were presented in short blocks, or “mini-blocks”, of 12 trials per encoding condition. Only one stimulus type (i.e., word pair or single word) was presented within a mini-block. An instruction prompt displayed “Get ready for the Imagery/Sentence task” for 4000 ms between mini-blocks.

At study, there were two encoding conditions. In the *visual imagery* condition, participants were instructed to generate an image, or token, representing the word or pair of words for each trial (e.g., Imagining an Olympic athlete for the word “winner”). For word pairs, participants were instructed to visualize tokens representing both words, and then imagine the two tokens interacting (e.g., An interview taking place at the Grand Canyon, for the pair “Panorama-Interview”). Visual imagery instructions explicitly noted that the two tokens had to be interacting to be considered a successful trial. In the *sentence generation* condition, participants were instructed to generate a sentence for the word or pair of words presented on that trial (e.g., The accomplice helped the thief escape, for the word “accomplice”). For word pairs, task instructions emphasized that both words had to be included in a single, meaningful sentence to be considered successful (e.g., The painter showed fatigue while producing the work of art, for the pair “Art-Fatigue”). In both study conditions, participants rated their success at generating a mediator (yes/no) on that trial. All yes/no responses were made with the index and middle fingers, respectively, of the right hand. Trials with no responses or more than one response as well as trials with response times less than 200 ms were excluded from the behavioral and neuroimaging analyses. Overall, encoding lasted approximately 45 minutes.

Immediately following the study session, memory for the word pairs seen at study was tested over 2 retrieval runs. Retrieval consisted of a 3-alternative forced choice recognition test that assessed memory for all 192 studied word pairs. Ninety-six studied pairs (half from each encoding condition) were tested per run. For each trial, a single cue word presented as part of a pair at study was displayed in the middle of the screen in white 36-point Arial font on a black background. Directly below the cue, three other words were simultaneously presented: the target (the correct pair), a rearranged pair lure (a word paired with a different word at study), and a single word lure (an item seen as a single word at study). The cue and response choices were presented for 4500 ms followed by a 250 fixation (see **Figure 1**). The target and lures were presented within mode. That is, for a cue word studied in the visual imagery condition, all three response options for that trial were also encountered in the visual imagery condition. Across retrieval trials, the target, rearranged lure, and single word lure options appeared equally often in the left, middle, and right locations below the cue word. Participants were instructed to endorse the word that was originally paired with the cue word at study. Trials where the target was accurately endorsed were considered “hit” trials; trials where the rearranged pair or single word lures were incorrectly endorsed were considered “error” trials. Within each retrieval run, equal numbers of studied words from each of the four study runs were presented. Trial types (sentence generation, visual imagery) were presented in a pseudorandom order so that no more than five trials of the same type were presented consecutively. Data analysis was constrained to trials with one retrieval response. Trials with greater or fewer than one response as well as trials with response times less than 200 ms were excluded. Overall, retrieval lasted approximately 15 minutes.

Immediately following retrieval, participants were taken out of the scanner suite and given an additional post-test assessment. The post-test was administered to obtain a rating of the quality for each mediator generated at the time of study. During the post-test, participants were shown all 192 intact word pairs they studied at encoding. Post-test trials were self-paced and were shown in a pseudorandom order with the constraint that no more than five trials of the same type could be presented in a row (visual imagery, sentence generation). For each word pair, participants made two judgments: First, they reported whether they successfully generated a mediator (visual image or sentence) for that trial at the time of study (yes/no). Second, they then rated the quality of the generated mediator for that trial. Because our primary interest in the post-test rating was for the quality rating data from this first post-test question is not shown. For images, they rated the vividness of the generated image on a 1 to 4 scale (1 =  no image to 4 =  highly vivid, almost like perception). Similarly, for sentences, participants rated the quality of the generated mediator on a 1 to 4 scale (from a simple sentence to a highly elaborated sentence).

### fMRI Acquisition

Functional and structural images were acquired with a Siemens Trio 3T full body scanner (Siemens, Erlangen, Germany) using a 12-channel parallel imaging head coil. First, T1-weighted magnetization-prepared rapid gradient echo scans (MP-RAGE; TE = 4.52 ms, 256×256 FOV) were acquired in 160, 1-mm thick, sagittal slices to obtain high-resolution structural images. Second, t2*-weighted functional images were acquired using a gradient echo pulse sequence (TR = 2000 ms, TE = 30 ms, flip angle = 90°, 3-mm in-plane resolution), collected in 37 slices (interslice gap 17.5%) aligned to the anterior-posterior commissural line covering the entire cerebrum. A total of 320 volumes were collected during each study run and 237 volumes were collected during each test run.

### fMRI Analysis

Functional data were analyzed with Statistical Parametric Mapping (SPM8; Wellcome Department of Cognitive Neurology) in MATLAB (R2008a; The Mathworks Inc., Natick, MA). The first five volumes of each session were discarded to allow for equilibration effects. The remaining echo planar image (EPI) volumes were corrected for differences in slice acquisition time using the middle slice of each volume as the reference, and spatially realigned to the first acquired volume. The structural scan of each participant was coregistered to the mean EPI image produced from the realignment and subsequently segmented and normalized to the Montreal Neurological Institute (MNI) T1 average brain template. These normalization parameters were applied to all EPI volumes and the normalized EPIs were resliced to 3 mm×3 mm×3 mm resolution and then spatially smoothed using an 8-mm full-width at half-maximum Gaussian kernel.

Analyses of the functional data from the study and test phases, which were modeled separately, were carried out in two steps. In the first step, neural activity was modeled as a series of 2 second epochs at study and at test coinciding with onsets of the various event types and convolved with a canonical hemodynamic response function. Time courses were down-sampled to the middle slice to form the covariates for the General Linear Model (GLM). For each participant and session, 6 covariates representing residual movement-related artifacts, determined by the spatial realignment step, were included in the first level model to capture residual (linear) movement artifacts. For both the study and test phase GLMs, trials with no responses or more than one response as well as trials with reaction times under 200 ms were not modeled. Voxel-wise parameter estimates for all covariates were obtained by Restricted Maximum-Likelihood (ReML) estimation, using a temporal high-pass filter (cut-off 128 seconds) to remove low-frequency drifts. Intrinsic autocorrelations within each session were corrected by applying a first-order autoregressive [AR(1)] model. The data were also scaled to a grand mean of 100 over all voxels and scans [32].

Contrasts of the parameter estimates for each participant were submitted to the second stage of analysis treating participants as a random-effect. Separate ANOVA models were created for study and test periods that allowed us to examine common memory effects for visual imagery and sentence generation trials as well as memory-by-condition interactions. The 2×2 model for the study period included factors of Condition (visual imagery, sentence generation) and Memory (hits, errors [trials where the rearranged pair or the single pair lure were endorsed]). Analysis was restricted to successful trials were participants reported generating a mediator at the time of study (e.g., the “yes” trials at encoding.) Due to insufficient trial numbers, the trials that were given a “no” response at encoding (e.g., encoding trials where a mediator was not successfully generated) were included in the model as regressors of no interest, but were not included in any planned contrasts. Further, given our principle focus on associative memory in this experiment, the single word trials at study were included in the model, but were not compared in any of the planned comparisons. Sixteen covariates modeling the mean across conditions for each participant were also added to each model, to remove between-subject variance of no interest. Statistical Parametric Maps (SPMs) were created of the T-statistics for the various ANOVA effects of interest, using a single pooled error estimate for all contrasts, whose nonsphericity was estimated using ReML as described in Friston et al. [33].

In both the study and test phases, the primary contrast of interest was between the “successful” hit and error trials, allowing us to examine associative memory accuracy effects for those trials where a mediator was successfully generated. Because of our interest in this uni-directional contrast (i.e. hits > errors), we report the results from one-tailed t-contrasts, thresholded at p<0.001, uncorrected, with a minimum cluster size of 5 contiguous voxels. Inclusive masking was carried out using a threshold of p<0.01 for the mask. Inclusive masks were applied to determine the overlap between regions associated with task-specific processing (regardless of memory judgment) and task-dependent memory effects. Exclusive masking was carried out using a liberal uncorrected threshold of p<0.05 for the mask. Exclusive masking was applied to identify regions showing associative memory effects common to both visual imagery and sentence generation, masking out the interactions between conditions. Both masked and unmasked contrasts were evaluated under a one-tailed uncorrected threshold of p<0.001 and a minimum cluster size of 5 contiguous voxels. Where noted neural activity for these peak maxima were plotted for the mean difference between the hit and error trials for the visual imagery and sentence generation conditions. Neural activity for these peak voxels reflects the parameter estimates for the convolved regressors and is presented in arbitrary units.

## Results

### Behavioral

Participants successfully generated mediators at the time of study more often for the sentence generation (91%, *SD* 8%) than for the visual imagery condition (82%, *SD* 10%), t(15)  = 5.32, p<.01). Overall hit rates and the corresponding reaction times, regardless of mediator success at encoding, are shown in **Table 1A**. Given the three-alternative forced choice task, chance performance with unbiased guessing would yield a 0.33 proportion of correct responses. Pairwise comparison showed no difference between hit or error rates between the encoding conditions, *t’*s <1. Response times between hits or errors also did not differ between the encoding conditions, *t*’s <1. We further calculated probabilities of hits and errors conditionalized on whether a mediator was reported as successful at the time of encoding (e.g. *p*(Visual Imagery Hit rate| Visual Imagery success rate), *p*(Visual Imagery Error rate|Visual Imagery success rate), etc.) (See **Table 1C**). A Memory (hit, error) by Success (successful, unsuccessful) by Condition (visual imagery, sentence generation) ANOVA on the conditionalized responses showed no main effects, *F*’s <2.9, but did results in a Response X Mediator Success interaction, *F*(1, 12)  = 15.0, p<.01. This interaction resulted from a higher hit than error rate for trials when a mediator was reported as successfully generated. No Condition effects suggest that associative memory accuracy did not differ between the two encoding tasks.

**Table 1:** Hit rates regardless of mediator success are displayed as a function of study condition in (A).

A)		
Response type	Visual Imagery	Sentence Generation
	Mean (SD)	Mean (SD)
Hits	0.59 (0.09)	0.61 (0.11)
**B)**		
Response type	Visual Imagery	Sentence Generation
	Mean (SD)	Mean (SD)
Hits	3019 (234)	3043 (195)
Errors	3467 (194)	3428 (217)
**C)**		
Conditionalized response	Visual Imagery	Sentence Generation
	Mean (SD)	Mean (SD)
Hits given successful mediator	0.62 (0.11)	0.62 (0.11)
Hits given unsuccessful mediator	0.51 (0.16)	0.49 (0.22)

Test phase response times for hit and error rates regardless of mediator success are displayed as a function of study condition in (B). The probabilities of hits conditionalized on the success of generating a mediator at encoding are displayed as a function of study condition in (C).

### fMRI

#### Analysis overview

There were three primary fMRI analyses performed on the functional data. First, we examined the main effects of encoding task at study regardless of memory outcome (i.e. across hits and errors) for trials with successfully generated mediators to determine regions associated with each respective task. Second, we examined *task-selective* regions showing associative memory effects specific to the visual imagery and the sentence generation conditions, respectively, at both study and test. Third, we examined *task-invariant* regions supporting associative memory shared by both the visual imagery and sentence generation conditions at both study and test.

### Main Effect of Encoding Condition

**Figure 2:**
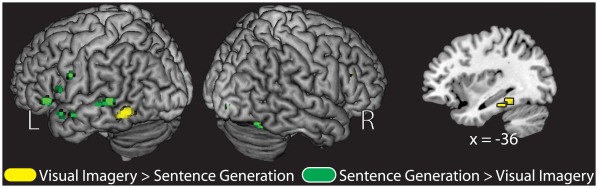
Main effect of instructed encoding strategy at study for trials with implementation success collapsed across subsequent associative memory judgments. Regions showing the effect of visual imagery (yellow; Visual Imagery > Sentence Generation) and the effect of sentence generation (green; Sentence Generation > Visual Imagery) are depicted. All regions displayed on a standard surface-rendered brain in MNI space. The left medial temporal lobe is shown through a sagittal slice at x = −36. Statistical threshold: p<0.001, uncorrected, 5 contiguous voxels.

In the first fMRI analysis, we examined the main effects of encoding task for visual imagery (visual imagery > sentence generation) and sentence generation (sentence generation > visual imagery). We included all items with reported mediator generation success at study regardless of subsequent memory outcome (i.e., hit and errors). Regions showing greater activity for visual imagery included the left inferior temporal cortex (BA 37), the left fusiform/posterior parahippocampus (BA 20), and the left inferior frontal gyrus (BA 45) (See **Table 2A** and **Figure 2**). In contrast, regions showing greater activity for sentence generation included bilateral inferior frontal regions (BA 47/48), the right lingual gyrus (BA 18), the left middle temporal gyrus (BA 21), as well as the left temporal pole (BA 38) (See **Table 2B** and **Figure 2**).

**Table 2:** Regions showing the task main effect at the time of study for the visual imagery condition (A) and the sentence generation condition (B).

Contrast	Region	Hemisphere	MNI Coordinates	BA	T-value	Cluster Size
A. Visual Imagery > Sentence Generation						
	Inferior Temporal	Left	−54 −55 −8	37	4.50	64
	Fusiform/Posterior Parahippocampus	Left	−36 −28 −20	20	3.96	7
	Inferior Temporal	Left	−36 −40 −14	37	3.75	12
	Inferior Frontal	Left	−42 29 10	45	3.57	8
B. Sentence Generation > Visual Imagery						
	Lingual	Right	21 −85 −5	18	4.44	24
	Inferior Frontal (orbital)	Left	−51 26 −2	47	4.19	27
		Left	−54 11 10	48	3.66	9
	Middle Temporal Gyrus	Left	−54 −40 4	21	3.83	35
		Left	−51 −1 −14	21	3.57	7
	Postcentral gyrus	Left	−60 −1 28	43	3.75	13
	Temporal pole	Left	−51 11 −14	38	3.59	11
	Pallidum	Left	−21 2 7	N/A	3.56	13

### Task-selective Associative Memory Effects

#### Study

**Figure 3:**
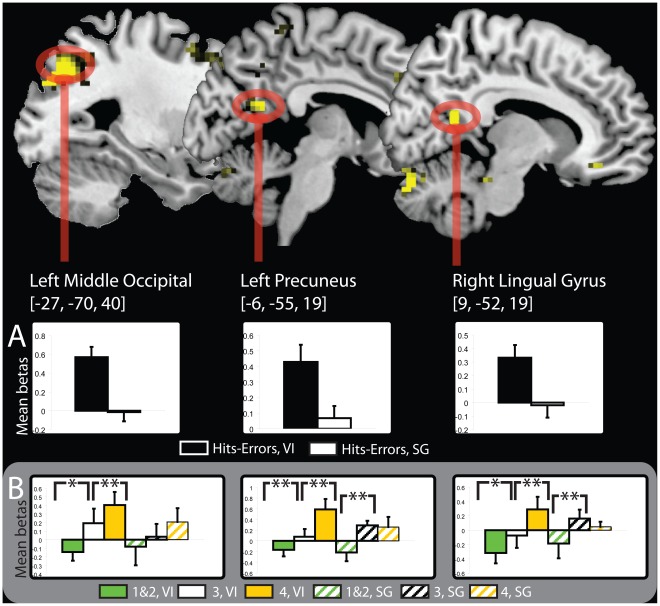
Task-selective activity at study. (A) Anatomic overlays show associative memory regions (hits > errors) exhibiting task-selective effects for the visual imagery condition (yellow colors). The graphs depict the parameter estimates of the peak voxels for three regions showing visual imagery-selective memory effects. In (A) the bars represent the difference between the betas for the hit and error trials for the visual imagery condition (black) and the sentence generation condition (white), respectively. In (B) the graphs represent activity, from left to right, for the left middle occipital gyrus, the left precuneus and the right lingual gyrus. The bars in each graph in (B) represent, from left to right, trials given a rating mediator quality rating of 1&2, 3, and 4 for the visual imagery condition, followed by trials given a mediator rating of 1&2, 3, and 4 for the sentence generation condition. Error bars depict the standard error of the mean. Whole brain statistical threshold: p<0.001, uncorrected, 5 contiguous voxels. ** = significantly different at p<.05, one-tailed; * = significance trend at p<.15, one-tailed. VI = visual imagery; SG = sentence generation.

As a second analysis, we examined task-selective associative memory effects for each respective task. We inclusively masked the subsequent memory effects for the visual imagery condition (hits > errors, visual imagery) with the interaction where the magnitude of the memory effect was greater for the visual imagery task than the sentence generation task ([Hits > Errors, visual imagery] > [Hits > Errors, sentence generation]) using a similar procedure as others [34], [35]. Task-selective regions that supported associative memory for the visual imagery condition in numerous regions throughout the cortex including the left middle occipital gyrus (BA 7), the left precuneus (BA 30), bilateral middle frontal gyrus (BA 11/47), the right lingual gyrus (BA 18) and the right fusiform/parahippocampus (BA 37), *(*See **Table 3A** *).* Effects for the left middle occipital, the left precuneus, and the right lingual gyrus are depicted in **Figure 3**. The comparable analysis for the sentence generation condition at the time of study yielded no regions supporting task-selective memory effects.

**Table 3:** Regions showing task-selective memory effects for the visual imagery (A) and for the sentence generation condition (B) at the time of *study*.

Contrast	Region	Hemisphere	MNI Coordinates	BA	T-value	Cluster Size
A. Task-selective, Visual Imagery, Study						
	**Middle Occipital**	Left	−27 −70 40	7	4.96	532
	**Precuneus**	Left	−6 −55 19	30	4.63	17
	Middle Frontal (orbital)	Bilateral	−30 41 5	11	4.43	50
			33 50 −8	47	3.89	18
	Angular	Right	27 −61 46	7	4.40	74
	Superior Frontal	Left	−18 20 58	8	4.23	167
	Middle Temporal	Left	−45 −25 −11	20	4.23	7
	Superior Frontal	Left	−12 50 10	32	3.91	7
	Supramarginal	Right	45 −34 43	2	3.88	10
	Superior Medial Frontal	Left	−9 29 37	32	3.87	19
	**Lingual**	Right	9 −52 19	18	3.78	16
	Anterior Cingulate	Right	9 35 −14	11	3.77	26
	Fusiform/Parahippocampus	Right	36 −37 −17	37	3.75	7
	Superior Medial Frontal	Left	0 62 19	10	3.68	17
	Precuneus	Left	−6 −43 64	5	3.39	11
B. Task-selective, Sentence Generation, Study						
	None					
C. Task-selective, Visual Imagery, Test						
	Anterior Hippocampus	Left	−21 −7 −20	N/A	6.58	168
	Putamen	Left	−27 −7 −5	N/A	4.74	
	Superior Temporal	Left	−45 −25 13	48	4.48	
	Medial Prefrontal (dorsal)	Left	−6 −56 7	17	6.02	61
	Superior Frontal	Left	−6 47 19	32	5.57	
	Anterior Cingulate	Left	−6 47 13	32	4.54	
	Medial Prefrontal (ventral)	Left	−3 59 −11	11	5.54	37
	Temporal Pole	Left	−36 17 −20	38	5.32	18
	Insula	Left	−36 20 −32	47	4.29	
	Superior Frontal	Left	−12 38 46	9	5.30	9
	Supramarginal	Left	57 −31 34	2	4.99	87
	Angular	Right	54 −52 31	40	4.11	
	Caudate	Left	−12 14 13	N/A	4.90	25
	Superior Frontal	Left	18 59 31	9	4.82	56
	Anterior Prefrontal	Left	−18 68 16	10	4.81	7
	Thalamus	Left	−6 −22 1	N/A	4.71	13
	Inferior Parietal	Left	−57 −52 37	40	4.69	25
	Middle Temporal	Left	−51 −43 −2	21	4.68	15
	Temporal Pole	Right	51 14 −20	38	4.52	8
	Middle Temporal	Right	60 −28 −8	21	4.49	41
	Superior Frontal	Left	−15 29 58	8	4.42	5
		Left	−21 17 49	8	4.34	14
	Cerebellum	Left	−3 −58 −2	N/A	4.34	17
	Hippocampus	Right	24 −10 −11	N/A	4.29	22
	Superior Temporal	Right	66 −16 13	22	4.19	16
	Precentral	Right	42 −22 61	4	4.18	27
	Middle Cingulate	Left	−3 −40 46	23	4.06	7
	Insula	Right	39 20 −14	38	4.02	7
	Hippocampus	Left	−33 −7 −26	N/A	4.01	5
	Insula	Right	39 5 7	48	3.95	13
	Postcentral	Left	−60 −7 16	48	3.94	7
	Insula	Left	−39 2 10	48	3.90	9
	Inferior Temporal	Left	−42 −10 −26	20	3.87	8
	Temporal Pole	Right	33 14 −16	38	3.75	14
D. Task-selective, Sentence Generation, Test						
	None					

Regions showing task-selective memory effects for the visual imagery (C) and for the sentence generation condition (D) at the time of *test*. Task-selective effects were defined by inclusively masking memory effects for each condition respectively (e.g., Hits>Errors, visual imagery) with the interaction where the memory effect was larger for one condition relative to the other (e.g., ([Hits>Errors, visual imagery] > [Hits>Errors, sentence generation]). Regions are listed from highest to lowest *t*-value. Regions listed without a cluster size are subsumed by the larger cluster listed directly above. Regions listed in bold are depicted in Figure 3. BA: Brodmann area.

To test whether brain regions active during the generation of sentences and visual images (i.e., task main effects) also contributed to successful memory, we further evaluated the task-selective memory effects by *constraining* them to those that overlapped with regions showing the task main effects as others have done [34]. To perform this analysis, we inclusively masked the task-selective memory effects (as performed directly above) with the relevant encoding task main effect (e.g., visual imagery>sentence generation). No voxels survived this additional analysis for either encoding task.

As an additional analysis, we examined functional activity in task-selective visual imagery memory regions as they related to the post-test vividness rating in a subset of participants (n = 6) that had a sufficient distribution of post-test vividness responses. Specifically, we selected a subset of participants who had enough “hit” trials (>10) receiving low (1&2 responses), medium (3), and high (4) ratings of vividness from the post-test to meaningfully compare the functional recruitment as a function of these ratings. We tested the prospect that task-selective memory regions would show a monotonic increase in activation related to increasing vividness ratings of the generated interactive image. To perform this analysis, we extracted parameter estimates from the subset of participants in the peak voxels isolated from all participants in the left middle occipital, the left precuneus, and the right lingual gyrus showing task-selective visual imagery effects at study (MNI coordinates, respectively  =  [−27, −70, 40]; [–6, −55, 19]; [9, −52, 19]). Comparisons between the low vividness trials, the medium vividness trials, and the high vividness trials, confirmed a monotonic increase in activity in the left precuneus (see **Figure 3B**) for the visual imagery trials (*t*’s >2.0, p<.05, one-tailed). No such pattern was found for sentence generation in this region. Trends in the same direction were also observed in the left middle occipital gyrus and the right lingual gyrus for visual imagery trials.

#### Test

Using the same procedure as described above for study, visual imagery-selective memory effects were found in multiple frontal, temporal and parietal regions including the left anterior hippocampus, the left medial prefrontal cortex (BA 11/17), and the left inferior parietal lobule (BA 40) (See **Table 3C**). The same procedure for the sentence generation condition yielded no regions showing sentence generation-selective memory effects.

We further examined regions showing task-selective memory effects at the time of test that were *constrained* by the encoding task main effects by inclusively masking the task-selective effects with the relevant encoding task main effect (e.g., visual imagery>sentence generation). No regions survived this masking procedure in either condition. We further examined functional activity at the time of test in task-selective memory regions as they related to the vividness rating in the subset of participants that had sufficient post-test vividness ratings to again examine monotonic increases in activation related to the vividness self-reports. No such effects were found in any of the test phase task-selective regions.

Following an analogous procedure to that of Johnson & Rugg [16], we performed an additional analysis to examine reinstatement effects unique to the visual imagery and the sentence generation conditions, respectively. We performed this analysis to determine whether study phase activity in support of memory was “reinstated” at the time of test. To find reinstatement effects for the visual imagery condition, we inclusively masked the memory effects at the time of study (hits>errors, visual imagery) with the memory effects at the time of test (hits>errors, visual imagery) with the task main effect at the time of study (visual imagery > sentence generation, study). This analysis yielded no effects for either the sentence generation or the visual imagery conditions.

### Task-invariant Associative Memory Effects

#### Study

**Figure 4:**
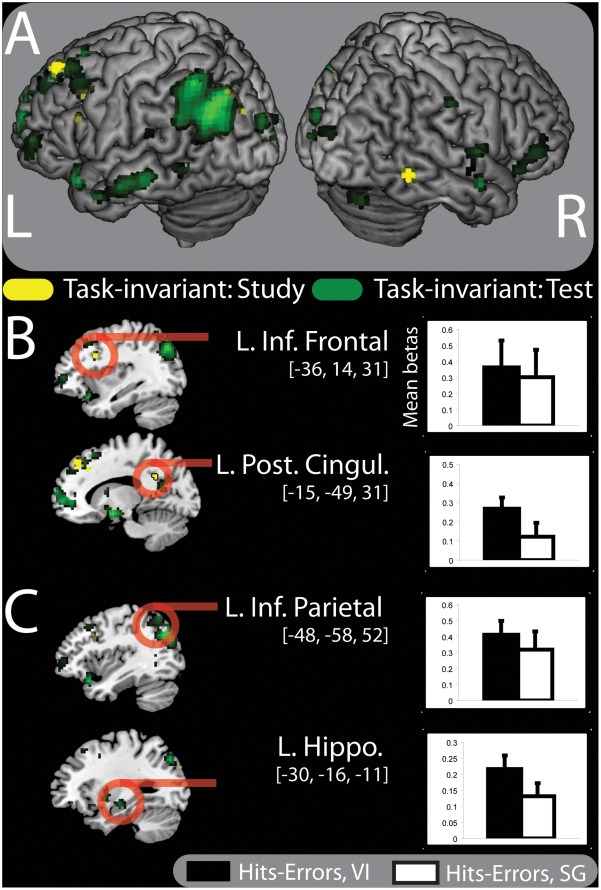
Task-invariant activity at study and test. (A) Associative memory regions (hits > errors) exhibiting task-invariant activation shared by both the visual imagery and sentence generation strategies at the time of study (yellow) and at the time of test (green) rendered on a standard brain in MNI space. Anatomic overlays and graphs depict the difference between the betas for the hit and error trials for the visual imagery condition (black) and the sentence generation condition (white), respectively, at the time of study (B) and at the time of test (C). Error bars depict the standard error of the mean. Statistical threshold: p<0.001, uncorrected, 5 contiguous voxels. L. Inf. Frontal  =  Left Inferior Frontal; L. Post. Cingul.  =  Left Posterior Cingulate; L. Inf. Parietal  =  Left Inferior Parietal; L. Hippo.  =  Left Hippocampus.

As a third analysis, we examined regions showing task-invariant subsequent memory effects common to both encoding tasks. To perform this analysis at study, we exclusively masked (see methods) the subsequent memory effects (hits> errors, collapsed over task) with the memory by task interaction. Results showed task-invariant memory effects in the left posterior cingulate (BA 23), the left superior frontal (BA 9), the right inferior temporal (BA 37), and the left Inferior frontal gyrus (BA 48) (See **Table 4A** and **Figure 4A**). Mean parameter estimates (betas) extracted from the peak voxel coordinate for the left inferior frontal gyrus and the left posterior cingulate are shown in **Figure 4B**.

**Table 4:** Regions showing task-invariant source accuracy effects at study (A) and test (B).

Contrast	Region	Hemisphere	MNI Coordinates	BA	T-value	Cluster Size
A. Task-invariant, study						
	**Posterior Cingulate**	Left	−15 −49 31	23	4.69	15
	Superior Frontal	Left	−15 41 46	9	4.12	39
	Inferior Temporal	Right	57 −49 −8	37	3.82	15
	Putamen	Right	30 8 22	N/A	3.91	7
	**Inferior Frontal**	Left	−36 14 31	48	3.60	32
	Calcarine	Left	−15 −49 10	30	3.74	27
	Middle Occipital	Left	−30 −88 31	19	3.40	10
	Inferior Frontal	Left	−54 17 25	44	3.36	5
B. Task-invariant, test						
	L Amygdala	Left	−18 −1 −14	34	6.48	282
	L Putamen	Left	−21 8 −8	48	5.64	
	L Temporal Pole	Left	−39 20 −20	38	5.56	
	L Superior Medial Frontal	Left	−9 62 4	10	6.44	878
	L Anterior Cingulate	Left	−6 44 7	32	6.01	
	L Medial Prefrontal	Left	0 59 −5	10	5.72	
	L Middle Occipital	Left	−39 −70 37	19	6.07	586
	L Angular	Left	−45 −70 31	39	6.00	
	**L Inferior Parietal Lobule**	Left	−45 −58 52	39	5.58	
	L Precuneus	Left	−3 −61 28	23	5.7	874
		Left	−9 −58 19	23	5.64	
		Left	−6 −64 43	7	4.88	
	R Putamen	Right	27 −1 −5	48	4.81	
	B Middle Temporal	Bilateral	60 −1 −17	21	4.72	9
			−60 −28 −11	20	4.69	96
	L Cuneus	Left	−6 −94 19	27	4.56	27
	**L Hippocampus**	Left	−30 −16 −11	N/A	4.47	26
	R Superior Temporal	Right	63 −4 7	48	4.19	21
	R Postcentral	Right	51 −19 37	3	4.08	11
	R Cerebellum	Right	39 −76 −29	N/A	4.01	19
	R Inferior Frontal	Right	54 38 1	45	3.98	67
	Calcarine		0 −100 7	17	3.96	12
	L Inferior Temporal	Left	−48 −55 −5	37	3.93	26
	R Cuneus	Right	18 −91 13	18	3.75	20
	R Insula	Right	42 2 7	48	3.68	12

Task-invariant regions were defined by exclusively masking subsequent memory regions (Hits>Errors) with regions showing the memory by condition interaction. Regions are listed from highest to lowest *t*-value. Regions listed without a cluster size are subsumed by the larger cluster listed directly above. Regions listed in bold are depicted in Figure 4. BA: Brodmann area.

#### Test

We further assessed task-invariant activity at the time of test using an analogous procedure to that used at study. Results indicated multiple regions showing task-invariant memory effects including the left hippocampus, the left medial prefrontal cortex (BA 10), and the left inferior parietal lobule (BA 39) (See **Table 4B** and **Figure 4A**). Extracted parameter estimates for the left hippocampus and the left inferior parietal lobule are shown in **Figure 4C**.

## Discussion

The primary goal of this investigation was to examine task-selective associative memory effects at the time of study and test for two encoding strategies known to facilitate memory for inter-item associations. In this experiment, we present three primary findings: first, we observed task-selective memory effects for visual imagery at the time of study and test in regions previously associated with visual imagery production including the precuneus and the middle occipital gyrus. No such effects were found for sentence generation at either study or test. Second, a subset of the regions exhibiting visual imagery-selective memory effects during study further exhibited activity that was positively related to post-scan self-reported vividness of the generated image. Third, we report task-invariant memory activity, common to both encoding tasks, at the time of study and test in regions previously associated with task-invariant memory including the left inferior frontal gyrus, the left posterior cingulate and the hippocampus.

### Task Main Effects at Study

The encoding tasks used in this experiment were effective in eliciting distinct patterns of activity for visual imagery and sentence generation, respectively, suggesting that the processes engaged by these two encoding tasks were distinct. Specifically, visual imagery engaged inferior temporal regions consistent with previous studies in which participants generated visual mental images [17], [19], [36], whereas sentence generation engaged lateral temporal and inferior frontal regions consistent with the semantic demands of the task as others have shown [7], [14], [20]. These data provide neuroimaging evidence to suggest that participants engaged in distinct encoding processes while generating mental images versus sentences.

### Task-selective Associative Memory Effects

Our primary interest in this investigation was to examine task-selective regions in support of memory for two encoding strategies known to facilitate paired associate memory–visual imagery and sentence generation. Previous investigations that have examined functional recruitment for different encoding strategies typically examine, directly or indirectly, levels-of-processing effects [1] by pitting elaboratively weak (i.e., shallow encoding) against elaboratively robust strategies (e.g., deep encoding) [7], [37]–[39]. Logan et al., for instance, compared a shallow semantic task (judging whether the first and last letters of a word were the identical) with a deep encoding task (concrete/abstract decision for words). Functional differences across such tasks could reflect differences in encoding operations but could also be due to differentially effective encoding operations (i.e., better memory). To truly isolate the neural substrates of specific encoding strategies it is useful to contrast tasks where baseline difference in memory performance do not influence the functional results such as in the current investigation.

In this investigation, we found task-selective memory effects at study for visual imagery, but not for sentence generation. We observed imagery-selective memory activity at the time of study in the middle occipital cortex, the precuneus, and the lingual gyrus. These regions have previously been associated with memory for materials studied via imagery [15]. Critically, we limited the analyses in this investigation to only those trials where participants reported success in generating each respective mediator. Although previous studies have utilized self-reports to evaluate encoding task success [14], [15], they have done so by measuring the quality of the generated mediator, not whether participants reported success in generating mediators at the time of study. Thus, these previous studies do not exclusively isolate activity associated with the successful strategy implementation. Finding imagery-selective memory effects at the time of study in regions previously associated with visual imagery confirms the notion that brain regions support memory in accord with the encoding operations deployed [9].

Intriguingly, we found evidence that task-selective visual imagery regions were linked to the post-scan subjective ratings of imagery vividness. More specifically, we observed that activity in the left precuneus showed a monotonic increase in activity that was positively related to the subjective reports of mental imagery vividness. Similar trends were noted in the left middle occipital and the right lingual gyrus. This finding reveals a novel correspondence between task-selective memory activations and self-reports of generated mediator quality. Specifically, these data suggest that the task-selective visual imagery regions functioned to facilitate the production of enriched visual mental images.

At the time of test, we found task-selective memory effects for visual imagery, but not for sentence generation. Task-selective visual imagery effects were found in bilateral hippocampi, the left medial prefrontal cortex, and the left inferior parietal lobule. Finding memory accuracy effects at the time of test that are contingent on the types of mental operations performed at study is consistent with previous findings [8], [9]. Activity in the left inferior parietal lobule has been associated with retrieval of specific episodic details, typically associated with recollective-type judgments [40], [41]. Indeed, previous work has found that inferior parietal activations, especially along the intraparietal sulcus, show sensitivity to amount of information recollected, with greater retrieval engaging greater recruitment [41]. In the case of the present study, it is possible that participants were recollecting more details associated with visual imagery than sentence generation trials. Although memory differences were not evident between tasks, it is possible that non-criterial recollection [42] was greater for the visual imagery condition resulting in both the inferior parietal and hippocampal imagery-selective activations found in this study.

It is more of a challenge to interpret the finding of imagery-selective effects in the left medial prefrontal cortex given that this area is unlikely to be a substrate for imagery per se. Previous work suggests that recollection of visual images generated at study requires reconstruction of the visual image from the target word [43]. Recent research has placed great focus on cognitive processes associated with memory reconstruction and the neural substrate implementing those processes [44]–[47]. Work from this perspective, suggests, notably, that a network of regions including the hippocampus and the medial prefrontal cortex are vital to this reconstruction process. While speculative, it is plausible that imagery-based reconstruction at the time of test would result in task-selective effects for pairs processed in the visual imagery condition in both the hippocampus and the medial prefrontal cortex. More “reconstruction” associated with the visual imagery task might further explain why reinstatement effects were not observed, at least for the visual imagery condition. If participants were effortfully engaged in reconstructing the episode rather than simply reinstating the perceptual details, few regions, if any, would survive the analysis to uncover reinstatement regions. As with all null results, this finding should be approached cautiously.

Although we found imagery-selective effects at study and test, we did not observe task-selective effects for sentence generation. There could be a number of different explanations for this outcome. One possibility is that both the sentence generation and visual imagery conditions involved overlap in the amount of semantic processing engaged by each respective task. We carefully selected materials that were not easily imagined, so that only implementing the instructed visual imagery strategy–using imageable tokens to represent abstract concepts–would be likely to engage imagery processes. However, it is possible, if not likely, that participants engaged in semantic processing during both tasks with the abstract word pairs (e.g., evaluating the meaning of each word while generating images and sentences). Recruitment of semantic processing in both instructed strategies would have reduced the likelihood of finding task-selective effects for the sentence generation condition. Our finding of task-selective effects for only one of our two tasks (imagery) diverges from previous investigations that have found task-selective effects for different types of encoding tasks [10], [11]. Otten et al., [11], for instance, identified task selective memory activations during study for both a semantic task (an animacy judgment) and a syllable counting task. One possible account for our discrepant findings compared to Otten [11] is that the tasks used in that study may have involved sufficiently different mental operations (judging animacy versus counting syllables) than the tasks used in this experiment. It should be noted however, that the task-selective masking procedure used to identify task specific memory effects in this investigation was also more conservative (i.e., used a more conservative threshold) than the procedure undertaken by Otten [11].

Another possible reason why we only observed task-selective memory effects for visual imagery is consistent with cognitive theory. Dual Coding Theory suggests that materials studied pictorially are better remembered than are materials studied only semantically [4], [48], [49]. According to Dual Coding Theory, this effect–the so-called picture superiority effect–results from engagement in both semantic and perceptual processing when studying visually complex materials. Although there were no overt images used as stimuli in this experiment (i.e., pictures), the mental images that participants conjured could be sufficient to afford this processing advantage for visual imagery. That is, if imagery generation involved a degree of semantic as well as additional imagistic processes, it is possible that the memory representations for the visual imagery task would be more detailed than those from the sentence generation condition. It may be the case, then, that the memory test used in this experiment (3-alternative forced choice) was not sufficiently sensitive to capture possible memory differences between encoding strategies, and that more sensitive memory measures such as recollection/familiarity judgments might help to clarify potential memory differences between sentence and visual imagery generation.

### Task-invariant Associative Memory Effects

In addition to the task-selective effects, we found task-invariant associative memory activity in the left inferior frontal gyrus and the left posterior cingulate at study, and the hippocampus and left inferior parietal lobule at test. A great deal of attention has been given to regions that contribute to memory accuracy regardless of stimulus domain or task [8], [25], [50], [51]. Lateral frontal, medial and lateral parietal, and the hippocampus, have all been previously implicated as part of an important episodic memory network [31], [52]. Previous work suggests that the hippocampus is important for inter-item binding and for the retrieval of those bound representations [26] in paired associate tasks [20] such as in the present study. As predicted, hippocampal activity at the time of test supported associative memory retrieval for materials processed with either encoding strategy, consistent with prior reports [8]. Regions of the lateral prefrontal cortex, especially those of the left inferior frontal gyrus, have been associated with elaborative encoding [29], [53]. Indeed, some evidence suggests that the inferior frontal gyrus, the hippocampus and the medial parietal cortex are part of a network that is involved in the generation and binding of associations into long-term memory [44]. Given the relatively robust memory performance for the trials associated with strategy success, the contributions of these regions supported memory for materials studied under either strategy. Further, lateral frontal activity, especially that of the inferior frontal gyrus has typically been associated with semantic processing [54]–[56]. We interpret task-invariant activity in the inferior frontal gyrus as a reflection of the shared semantic processing across both tasks. Such an explanation supports the notion that participants were engaging in semantic processing when generating both visual images and sentences in this experiment.

Our observation of activations in both task-invariant and task-selective memory regions is consistent with previous work that suggests that different regions operate to support memory in dynamic manner. Indeed, previous work suggests that some regions of the brain support memory in a domain-general fashion regardless of task set [8], [25], [26] while other regions seem to support memory in a task-dependent manner [7]–[11]. Indeed, these data and the work of others suggests that the contributions of both task-selective and task a-specific regions make important contributions to the formation of memory.

### Conclusions

In this experiment, we found evidence of task-selective associative memory effects at the time of study and test for visual imagery. No such effects were found for sentence generation, suggesting that both visual imagery and sentence generation shared overlapping semantic processing, but that visual imagery additionally engaged imagistic processes. We further found evidence that the task-selective visual imagery regions were linked to self-reports of the vividness of the generated mediator in a subset of participants, suggesting that activity in imagery-selective memory regions covaried with the vividness of the generated visual image. Overall, this experiment provides evidence to support the notion that regions of the brain operate in support of memory at the time of study and test in according with the mental operations successfully engaged at the time of study.
